# GRANDPARENTS’ ACTIVITY ENGAGEMENT WITH GRANDCHILDREN AS A FUNCTION OF CAREGIVER STATUS

**DOI:** 10.1093/geroni/igad104.0856

**Published:** 2023-12-21

**Authors:** Abigail Stephan, Athena Chung Yin Chan

**Affiliations:** Clemson University, Clemson, South Carolina, United States; Texas Tech University, Lubbock, Texas, United States

## Abstract

Though the grandparent population varies across virtually all sociodemographic characteristics, caregiver status (i.e., whether grandparents are raising grandchildren), may be most salient in how grandparents engage in activities and perceive their roles in relation to their grandchildren. Informed by the bioecological process-person-context-time model, this mixed-methods study explores how activity engagement varies by caregiver status and whether activity engagement with grandchildren is related to grandparents’ perceived roles. A convenience sample of 86 mid-life and older adults (Mage=65.12 years, range=42-82) completed an online survey with open- and closed-ended items related to their grandparenting experience. 22% of grandparents self-identified as grandparent caregivers (GC), or those who have been raising their grandchildren for six months or longer; the remaining 78% self-identified as non-caregiving grandparents (NCG). Comparative analyses did not yield statistically significant results, revealing GCs and NGCs largely engaged in similar activities with their grandchildren. However, activity engagement intervals differed by group (i.e., daily basis for GCs vs. weekly or monthly basis for NGCs), and a greater proportion of NCGs reported digital activity engagement with their grandchildren. Both GCs and NGCs identified their roles as emotional supporters to their grandchildren, though GCs also identified with instrumental roles (e.g., surrogate parent, anchor, financial supporter). These findings shed light on the connection between self-reported activity engagement and subjective role perceptions across just one sociodemographic characteristic—caregiver status—and suggest both groups share a similar grandparenting foundation, with an additional, “dual-roles” component for GCs. Implications for future research and practice will be discussed.

